# Supplementary material for: The first Miocene fossils of Lacerta cf. trilineata (Squamata, Lacertidae) with a comparative study of the main cranial osteological differences in green lizards and their relatives
Source: PLoS One. 2019 Aug 21;14(8):e0216191. doi: 10.1371/journal.pone.0216191 (PMC6703700; doi:10.1371/journal.pone.0216191)
Supplement: S1 Dataset — The CT data links for Figshare and Morphosource. (DOCX) [file pone.0216191.s001.docx]

**Čerňanský and Syromyatnikova PLOS ONE**

**S1: Repository of computed tomography data**

**1. FIGSHARE https://figshare.com**

***Lacerta bilineata* NHMW 18599 DOI:** 10.6084/m9.figshare.8342702

***Lacerta bilineata* NHMW 35860-1 DOI:**10.6084/m9.figshare.8396990

***Lacerta bilineata* NHMW 35860-2 DOI:**10.6084/m9.figshare.8397113

***Lacerta media* NHMW 38408 DOI:** 10.6084/m9.figshare.8343257

***Lacerta media* NHMW 18956-1 DOI:**10.6084/m9.figshare.8397125

***Lacerta media* NHMW 18956-4 DOI:**10.6084/m9.figshare.8397161

***Lacerta pamphylica* NHMW 35861DOI:**10.6084/m9.figshare.8343413

***Lacerta pamphylica* ZSM 939/2005 DOI:** 10.6084/m9.figshare.8343836

***Lacerta pamphylica* ZSM 1047/2005 DOI:**10.6084/m9.figshare.8397191

***Lacerta strigata* NHMW 39765 DOI:**10.6084/m9.figshare.8343449

***Lacerta strigata* NHMW 10917-1 DOI:**10.6084/m9.figshare.8397404

***Lacerta strigata* NHMW 10917-2 DOI:**10.6084/m9.figshare.8397470

***Lacerta trilineata* NHMW 27665 DOI:** 10.6084/m9.figshare.8343542

***Lacerta trilineata* NHMW 26524-1 DOI:** 10.6084/m9.figshare.8397542

***Lacerta trilineata* NHMW 26524-2 DOI:** 10.6084/m9.figshare.8397575

***Lacerta agilis* NHMW 39028 DOI:** 10.6084/m9.figshare.8343695

***Lacerta agilis* NHMW 30885-1 DOI:**10.6084/m9.figshare.8396912

***Lacerta agilis* NHMW 36392-1 DOI:**10.6084/m9.figshare.8396981

***Lacerta schreiberi* NHMW 10809 DOI:** 10.6084/m9.figshare.8343716

***Lacerta schreiberi* NMHW 10808-1 DOI:**10.6084/m9.figshare.8397218

***Lacerta schreiberi* NHMW 10808-4 DOI:** 10.6084/m9.figshare.8397362

***Lacerta viridis* NHMW 40137-1 DOI:** 10.6084/m9.figshare.8343779

***Zootoca vivipara* NHMW 32438-1 DOI:**10.6084/m9.figshare.8396822

***Timon lepidus* NHMW 10921-1 DOI:** 10.6084/m9.figshare.8396852

***Podarcis muralis* NHMW 39359-1 DOI:**10.6084/m9.figshare.8396876

***Takydromus sexlineatus* DE 134 DOI:**10.6084/m9.figshare.8396960

***Meroles ctenodactylus* NHMW 31376-1 DOI:**10.6084/m9.figshare.8396951

***Gallotia stehlini* NHMW 11031-1 DOI:**10.6084/m9.figshare.8396888

***Psammodromus algirus* NHMW 36038-2 DOI:**10.6084/m9.figshare.8396897

**2. Morphosource**[**https://www.morphosource.org/**](https://www.morphosource.org/)

***Lacerta viridis* UF 65017 Occurrence ID:**a70ce909-ac0c-420c-ac0f-9ba4e9a5019e

**MorphoSource Identifier:** S12333
